# Infants with neonatal Chronic Lung Disease are associated with delayed auditory conduction in the rostral brainstem after term

**DOI:** 10.1016/j.clinsp.2024.100341

**Published:** 2024-03-07

**Authors:** Ze Dong Jiang, Cui Wang, James K. Jiang, Jin Wang

**Affiliations:** Division of Neonatology, Children's Hospital of Fudan University, China

## Abstract

**Aims:**

Very Low Birthweight (VLBW) infants with neonatal Chronic Lung Disease (CLD) have been found to have functional impairment of the brainstem auditory pathway at term. This study investigated the functional status of the brainstem auditory pathway in VLBW infants with CLD after term for any abnormality.

**Methods:**

Fifty-two VLBW infants were recruited at 50 weeks of Postconceptional Age: 25 with neonatal CLD and 27 without CLD. None had any other major complications to minimize confounding effects. Brainstem Auditory Evoked Responses were studied at 21‒91/s click rates.

**Results:**

Compared with those without CLD, VLBW infants with CLD had relatively shorter latencies of BAER waves I and III, associated with a slightly lower BAER threshold. Wave V latency and I‒V interpeak interval did not differ significantly between the two groups of infants. The I‒III interval in infants with CLD was shorter than in those without CLD at 91/s clicks. However, the III‒V interval was significantly longer than in those without CLD at all click rates (all *p <* 0.05). There were no significant differences in the amplitudes of BAER wave components between the two groups of infants.

**Conclusions:**

The main BAER abnormality in VLBW infants with CLD was a prolonged III‒V interval. Auditory conduction is delayed or impaired at more central regions of the brainstem in CLD infants. After term central auditory function is adversely affected by neonatal CLD. Monitoring post-term change is required to provide valuable information for post-term care of CLD infants.

## Highlights

• CLD infants had delayed auditory conduction at more central brainstem regions.
• Postnatal central auditory function is adversely affected by neonatal CLD.
• Monitoring post-term auditory change is warranted for managing CLD infants after term.

## Introduction

Poor neurodevelopmental outcome is a significant concern in infants with Very Low Birthweight (VLBW).^1^, ^2^, ^3^, ^4^ A major perinatal respiratory morbidity associated with VLBW is neonatal Chronic Lung Disease (CLD). It occurs predominantly in VLBW infants who are born very prematurely, with their lungs underdeveloped in the womb. Despite recent notable advances in neonatal care, CLD remains a very significant complication of preterm birth, often resulting in prolonged hospital stays and long-term morbidity. These infants have been found to have brain white matter damage and maturational delay in brain structure and function.^5^^,^^6^ Compared with infants without CLD, those with CLD are at an increased risk of developing neurological problems, such as significantly lower developmental quotients for adaptability, gross motor, fine motor, language, and social skills in early childhood.^7^, ^8^, ^9^

Previous studies with Brainstem Auditory Evoked Response (BAER) have shown that at-term VLBW infants with CLD had a significant functional abnormality in the brainstem auditory pathway.^10^ The BAER is a non-invasive and objective electrophysiological method to examine the functional integrity of the brainstem auditory pathway. During early life, the measurements of BAER variables primarily reflect nerve conduction velocity associated with axonal diameter, myelination, and synaptic function along the brainstem auditory pathway.^11^, ^12^, ^13^, ^14^ In infants with perinatal complications or problems that may involve the brainstem auditory pathway, this abnormality indicates brainstem auditory impairment and/or maturational delay, primarily related to delayed or impaired myelination of the pathway.^11^^,^^13^^,^^14^

Neonatal CLD, which occurs predominately in VLBW infants, and associated perinatal conditions, particularly chronic sublethal hypoxia, significantly affect the functional status of the immature central auditory pathway.^10^^,^^11^ At term VLBW infants with CLD were found to be associated with a significant increase in BAER wave V latency and I‒V and III‒V interpeak intervals, suggesting a major impairment in brainstem auditory function.^10^ Whether such abnormality in CLD infants extends beyond term remains to be studied. The understanding is important for post-term care and management of CLD infants to help improve their neurodevelopmental outcomes. It is presumable that the brainstem auditory impairment in CLD infants found at term would improve with age but there would still be some abnormalities after term. Thus, the authors carried out a BAER study in VLBW infants at 50 weeks of Postconceptional Age (PCA) to assess the functional status of the brainstem auditory pathway after term in VLBW infants with CLD. The results in infants with CLD were compared with those without CLD to investigate any functional abnormality in the pathway after term in CLD infants.

## Methods

### Study population

As previously described, based on the measurement of the I‒V interval, the most widely used BAER variable to reflect brainstem auditory function, in normal term infants and previous experience, and the power calculation (α = 0.05, β = 0.10), 16 infants are required in each group to achieve statistical significance for comparing between groups (probability < 0.05).^11^^,^^15^^,^^16^ This study recruited 52 VLBW infants who had a birthweight below 1500 g, including 25 infants who had neonatal CLD (CLD group) and 27 who did not have CLD (non-CLD group), were recruited from the Children's Hospital of Fudan University. The subject number in each group was greater than the required sample size to minimize any bias and more reliably analyze BAER data. The diagnostic criteria for CLD were the same as those previously described.^10^ These included the requirement for supplementary oxygen or ventilatory support beyond 36 weeks of Postconceptional Age (PCA) to maintain PaO_2_ > 50 mmHg, clinical signs of chronic lung respiratory disease, and radiographic evidence of CLD (persistent strands of density in both lungs). Of the 25 CLD infants, 14 were classified as mild, 6 moderate, and 5 severe, according to the diagnostic criteria described by Jobe and Bancalary.^17^ The subjects were all in stable clinical status at the time of BAER testing.

The subject's demographics at birth and main perinatal conditions or complications are presented in Table 1. To minimize any confounding effects the authors excluded those infants who had other major perinatal problems that may significantly affect the brainstem auditory pathway, mainly including grades III and IV intraventricular hemorrhage, intrauterine growth restriction, congenital malformation or chromosomal anomalies, congenital or perinatal infection of the central nervous system, syndromes, family history of hearing loss, severe perinatal hypoxia-ischemia, hyperbilirubinemia, sepsis, necrotizing enterocolitis.^11^^,^^12^^,^^15^ Prior to study entry, parental consent was obtained for each infant when they were at a mean PCA of 50 weeks. This cohort study followed the STROBE Statement.

**Table 1:** Subjects’ demographics at birth and main perinatal conditions.

Demographics	CLD	Non-CLD
Male/female (n)	9/16	9/18
Gestation (weeks)	25.8 ± 1.8^a^	27.7 ± 1.4
Postconceptional Age at BAER testing (weeks)	50.5 ± 3.9	51.2 ± 3.9
birthweight (g)	796 ± 185^a^	1060 ± 13
Apgar score at 5 min	8.1 ± 1.5	7.7 ± 1.6
Head circumference at birth (cm)	25.2 ± 2.3	25.8 ± 2.0
Head circumference at BAER testing (cm)	39.7 ± 2.1	38.6 ± 4.9

a *p <* 0.001 is the significance for the comparison of CLD and non-CLD infants.

### BAER recording and analysis

At the BAER testing, the PCA was almost the same in the CLD (50.5 ± 3.9 weeks) and non-CLD groups (50.4 ± 4.0 weeks). The protocols of recording were approved by the Ethics Committee of the Children's Hospital (JZDOX1211). In brief, surface electrodes were placed, respectively, at the middle forehead, and the left and right earlobe. Rarefaction clicks of 100 µs were delivered monaurally through an earphone and presented at three repetition rates to examine whether any abnormalities consistently exist at all click rates and whether there are any differences in the results between different click rates. The clicks were presented at 21/s, 51/s, and 91/s in the first run and a reverse sequence was used in the second run. All infants were tested with clicks at 60 dB normal Hearing Level (nHL). Five CLD infants had a BAER threshold greater than 20 dB nHL (> 20‒35 dB nHL), and were subject to higher click intensity to obtain BAER recording at a hearing level of 40 dB or slightly higher above the BAER threshold of individual infants to obtain well-formed BAER morphology for all infants. Evoked brain responses to 2480 clicks were preamplified and bandpassed at 100-3000 Hz.

### Data analysis

In both the CLD and the non-CLD groups, a detailed analysis of BAER data was carried out for the BAER recordings that were obtained at a hearing level of 40 dB or slightly higher above the BAER threshold of each infant. Analysis of BAER variables was conducted blind to the medical history and clinical data of the infants. The measurements from two replicated BAER recordings to each recording condition were averaged and analyzed statistically using an SPSS package version 22 (Chicago, IL).

The subjects’ data presented in Table 1 were compared between CLD and non-CLD groups. Pearson's χ2 statistic and Fisher's exact test were used for the comparison of any categorical variable (gender). The independent samples *t*-test was used for the comparison of continuous variables (gestation, PCA, birthweight, Apgar score, occipito-frontal circumference). A 2-tailed value of *p <* 0.05 was considered statistically significant. The one-sample Kolmogorov-Smirnov test showed that the latencies and amplitudes of BAER wave components and interpeak intervals all followed a normal distribution, but the V/I and V/III amplitude ratios did not. This was the same in the CLD and non-CLD groups. As such, for statistical comparison between the two groups of infants, the Student *t*-test was used for BAER wave latencies and amplitudes, and interpeak intervals, while the Mann-Whitney test was used for the V/I and V/III amplitude ratios. A 2-tailed value of *p <* 0.05 was considered statistically significant. Correlation analysis was conducted for the relationship between BAER variables and the repetition rate of clicks. Correlation coefficients (two-tailed test of significance) were then obtained.

## Results

### Comparison of subjects’ demographics between CLD and non-CLD groups

The gestation and birthweight in the CLD group were both significantly smaller than those in the non-CLD group (*p <* 0.001, 0.001) (Table 1). There were small differences in other demographic data between the CLD and non-CLD groups. However, none of the differences differed significantly (Table 1). In particular, the Postconceptional Age at BAER testing was similar in the two groups of infants.

### Comparison of BADER threshold and hearing level between CLD and non-CLD groups

The threshold of BAER, obtained at the time of testing, in the CLD group (10.2 ± 7.9 dB nHL) was slightly lower than in the non-CLD group (12.6 ± 6.5 dB nHL), which did not differ significantly. Most infants had a BAER threshold ≤ 20 dB nHL, so the BAER recordings obtained at 60 dB nHL clicks were used for analysis. For those with a BAER threshold > 20 dB nHL, the BAER data obtained at higher click intensities were used for analysis to achieve a hearing level of 40 dB or slightly higher: 70 dB nHL for the infants with a threshold > 20‒30 dB nHL (*n =* 4), and 80 dB nHL for the infants with a threshold 35 dB nHL (*n =* 2). Thus, all measurements of BAER components were obtained at a hearing level of 40 dB or slightly higher above the BAER threshold of each infant. The hearing level at which BAER data were analyzed was 51.8 ± 9.2 dB in the CLD group and 50.4 ± 6.5 dB in the non-CLD group, which did not differ significantly.

### Comparison of BAER wave latencies and intervals between CLD and non-CLD groups

Table 2 presents the measurements of BAER wave latencies and interpeak intervals for both the CLD and non-CLD groups and the results of statistical comparison between the two groups. Wave I latency was shorter in the CLD group than in the non-CLD group, which was true for all click rates of 21‒91/s (all *p <* 0.05). Similarly, wave III latency in the CLD group was consistently shorter than in the non-CLD group at all click rates (all *p <* 0.05). There were no significant differences in wave V latency between the two groups of infants, although the latency in the CLD group was slightly shorter than in the non-CLD group. This was the case at all click rates.

**Table 2:** Means and Standard Deviations (SD) of BAER wave latencies and interpeak intervals (≥ 40 dB above BAER threshold) in CLD infants and non-CLD infants and the results of comparisons between the two groups of infants.

BAER	Subjects	21/s	51/s	91/s
Variables		mean ± SD	mean ± SD	mean ± SD
I (ms)	Non-CLD	2.44 ± 0.25	2.57 ± 0.22	2.69 ± 0.22
	CLD	2.24 ± 0.37^a^	2.39 ± 0.40	2.52 ± 0.31^a^
III (ms)	Non-CLD	4.99 ± 0.28	5.18 ± 0.29	5.40 ± 0.28
	CLD	4.70 ± 0.35^a^	4.93 ± 0.48^a^	5.12 ± 0.41^a^
V (ms)	Non-CLD	7.03 ± 0.35	7.36 ± 0.36	7.77 ± 0.37
	CLD	6.86 ± 0.36	7.22 ± 0.39	7.61 ± 0.49
I‒V (ms)	Non-CLD	4.59 ± 0.35	4.79 ± 0.37	5.06 ± 0.37
	CLD	4.62 ± 0.25	4.83 ± 0.25	5.05 ± 0.27
I‒III (ms)	Non-CLD	2.54 ± 0.22	2.61 ± 0.20	2.70 ± 0.20
	CLD	2.44 ± 0.17	2.52 ± 0.20	2.56 ± 0.18^a^
III‒V (ms)	Non-CLD	2.04 ± 0.22	2.18 ± 0.23	2.35 ± 0.26
	CLD	2.18 ± 0.17^a^	2.32 ± 0.18^a^	2.50 ± 0.21^a^

a *p <* 0.05 is the significance for the comparison of CLD and non-CLD infants.

**Fig. 1:**
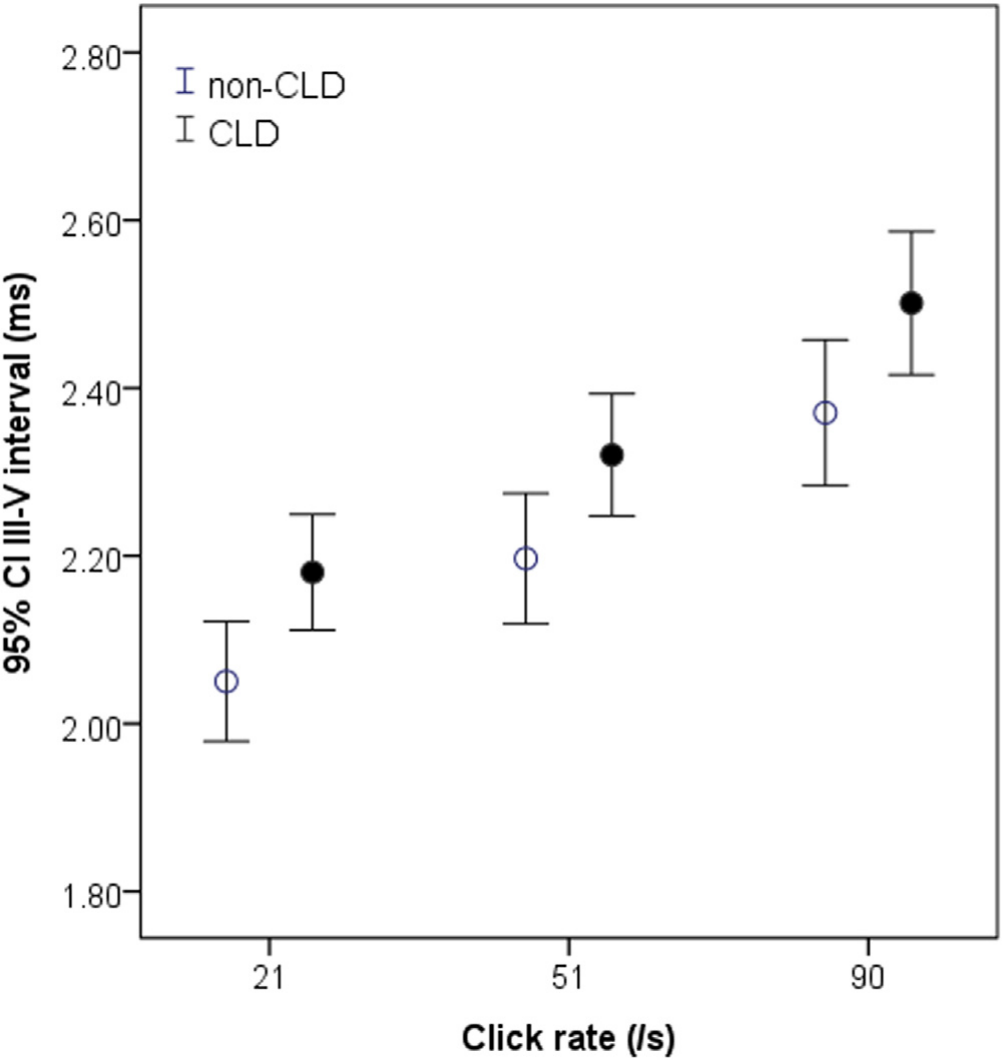
Measurements of the III‒V interval at 21‒91/s in non-CLD and CLD groups. The interval in CLD group (black circle) is significantly longer than in non-PD group (white circle) at all click rates, particularly at higher click rates. *p < 0.05 for comparison between CLD and non-CLD groups.

There were obvious differences between the two groups of infants in the I‒III and III‒V intervals (Table 2). The I‒III interval in the CLD group tended to be shorter and was significantly shorter than in the non-CLD group at the highest click rate of 91/s (*p <* 0.05) (Table 2). In contrast, the III‒V interval in the CLD group was significantly longer than that in the non-CLD group at all rates (all *p <* 0.05) (Table 2). This difference can be clearly seen graphically in Fig. 1. No significant difference was found in the I‒V interval between the CLD and non-CLD groups.

### Comparison of BAER wave amplitudes between CLD and non-CLD groups

The measurements of BAER wave amplitudes are presented in Table 3 for both the CLD and non-CLD groups. There were some differences between the two groups of infants in the amplitudes of waves I, III, and V at various click rates, but none of the differences reached statistical significance. This was also the case for the V/I and V/III amplitude ratios. No significant differences were found between the CLD and non-CLD groups at any click rate.

**Table 3:** Means and Standard Deviations (SD) of BAER wave amplitudes and amplitude ratios at term (≥ 40 dB above BAER threshold) in CLD and non-CLD infants and the results of comparisons between the two groups of infants.

BAER	Subjects	21/s	51/s	91/s
Variables		Mean ± SD	mean ± SD	mean ± SD
I (µV)	Non-CLD	0.168 ± 0.071	0.136 ± 0.057	0.146 ± 0.076
	CLD	0.187 ± 0.075	0.132 ± 0.043	0.130 ± 0.043
III (µV)	Non-CLD	0.208 ± 0.087	0.189 ± 0.080	0.155 ± 0.054
	CLD	0.228 ± 0.087	0.209 ± 0.065	0.162 ± 0.062
V (µV)	Non-CLD	0.227 ± 0.087	0.205 ± 0.070	0.173± 0.057
	CLD	0.247 ± 0.106	0.231 ± 0.072	0.193 ± 0.055
V/I	Non-CLD	1.508 ± 0.652	1.686 ± 0.808	1.404 ± 0.644
	CLD	1.315 ± 0.692	1.816 ± 0.647	1.614 ± 0.647
V/III	Non-CLD	1.223 ± 0.622	1.263 ± 0.777	1.177 ± 0.486
	CLD	1.058 ± 0.388	1.080 ± 0.347	1.287 ± 0.549

### Correlation of BAER wave components with the click repetition rate

As the repetition rate of clicks was increased, BAER wave latencies and interpeak intervals were all increased (Tables 1 and 2), whereas BAER wave amplitudes were all reduced (Table 3). This was true for both the CLD and non-CLD groups. The changes in BAER variables with varying click rates were generally similar for the two groups of infants, with only small differences. The latencies for BAER waves I, III, and V, and interpeak intervals were all correlated positively and significantly with the repetition rate of clicks in both the CLD and non-CLD groups (*r =* 0.25‒0.58, *p <* 0.05‒0.01). In contrast, the amplitudes of BAER waves I, III, and V were all correlated negatively and significantly with click rate in both the CLD and non-CLD groups (*r =* -0.28-0.71, *p <* 0.05‒0.01). There were small differences in the slopes of BAER variable rate-dependent functions between the two groups of infants, but none reached statistical significance. The V/I and V/III amplitude ratios varied slightly at different click rates. Neither was correlated significantly with click rates in the CLD and non-CLD groups.

## Discussion

At PCA 50 weeks the VLBW infants with CLD showed some differences in the BAER from the those without CLD. The main difference was a significantly longer III‒V interval, along with a relatively shorter I‒III interval, in the CLD infants. This was generally true at all click rates used. VLBW infants with neonatal CLD manifest a relatively poorer brainstem auditory function, mainly a delay in auditory conduction at more central or rostral regions of the brainstem.

In the previous study of BAER in VLBW infants at PCA 40 weeks, the wave V latency and I–V and III–V intervals were significantly increased in the infants with neonatal CLD.^10^ In the present study at PCA 50 weeks, the major finding is an increased III‒V interval in VLBW infants with CLD, compared with those infants without CLD. The III‒V interval, the second or later sub-component of the I‒V interval, reflects auditory conduction at more central regions of the brainstem auditory pathway.^11^^,^^12^^,^^18^ The increase in this interval in the CLD infants, reflecting auditory conduction delay, suggests impaired central regions of the pathway. After term auditory function at more central brainstem regions is still adversely affected by neonatal CLD.

Because of the multifactorial nature of the disease process, the impaired auditory function at more central brainstem regions in CLD infants could be related to various associated perinatal conditions and risk factors. In early life, BAER undergoes considerable rapid maturation with increasing age. Chronological age, i.e., PCA, at the time of BAER testing, is the most important physiological factor that significantly affects the measurements of BAER variables. Seethapathy and colleagues studied preterm infants born at a wide range of gestation.^19^^,^^20^ The results showed that gestational age at birth does not seem to influence BAER wave latencies and interpeak intervals at the same or similar PCA. Maturation of the brainstem auditory pathway occurs in a similar manner in preterm infants regardless of gestational age at birth. They conclude that preterm birth alone as a risk factor does not appear to have any marked effect on BAER development. In this study, the CLD infants had smaller gestation and birthweight, compared with the non-CLD infants. However, the PCA at BAER testing was similar in the two groups of infants. There were also no significant differences between the two groups in other demographics and perinatal conditions. It has been recognized that it is the associated perinatal conditions and/or complications of small gestation and low birthweight, rather than the gestation and low birthweight *per se*, which are the risk factors. A considerable difference in gestation and birthweight could exert a certain limited effect on the measurements of BAER variables.^21^ Nevertheless, the small differences in gestation (2-week difference only) and birthweight between this CLD and non-CLD groups cannot exert any marked effect on measurements of BAER variables, so long as the PCA is similar in the two groups of infants. Therefore, the difference in brainstem auditory function between VLBW infants with CLD and those without CLD is primarily attributed to neonatal CLD.

During early life, neural conduction along the brainstem auditory pathway is primarily and closely related to myelination along the pathway.^11^^,^^12^^,^^14^^,^^18^ An increase in the III‒V interval primarily reflects slower neural conduction due to delayed or impaired myelination of the central auditory pathway. In the CLD infants, the increase in the III‒V interval is primarily suggestive of delayed or impaired myelination in more central brainstem regions. This is comparable with previous magnetic resonance imaging findings in infants with CLD that neonatal CLD is strongly associated with an increased risk for brain white matter damage and delay in structural brain maturation.^22^

The pathophysiology of neurological impairment in neonatal CLD is complex and multifactorial.^9^^,^^23^ Among the others, the chronic sublethal hypoxia present in CLD infants plays a crucial role in brain damage and neurological impairments. During the course of CLD, infants often experience intermittent hypoxic episodes or frequent episodes of hypoxaemia. This results in chronic or prolonged sublethal hypoxia and the requirement of prolonged mechanical ventilation and oxygen therapy. Numerous investigators have shown evidence that acute lethal hypoxia, which is often associated with ischemia, occurring during the neonatal period, i.e., hypoxia-ischemia or perinatal asphyxia, seriously damages the immature brain, including the brainstem auditory pathway, even leading to neuronal death.^11^^,^^12^^,^^24^ The effect of chronic sublethal hypoxia occurring during the neonatal period on the immature brain and auditory pathway remains less well studied.^25^^,^^26^ Animal experiments revealed that chronic sublethal hypoxia significantly affects the immature brain, resulting in severe impairment in corticogenesis, a significant decrease in subcortical white matter, and a significant reduction in glia.^1^^,^^26^, ^27^, ^28^, ^29^ In VLBW infants, chronic sublethal hypoxia occurring during the neonatal period is typically seen in those who suffer neonatal CLD.^26^ Infants with CLD usually require supplemental oxygen or ventilatory support beyond PCA 36 weeks. The requirement often continues beyond the full-term period due to the persistent chronic hypoxia in many CLD infants.

The latency of BAER wave I reflects the functional status of the peripheral auditory pathway, and is significantly affected by BAER threshold.^11^^,^^12^ A lower BAER threshold and higher hearing level (i.e., the click intensity above the BAER threshold) at testing shortens the latencies of BAER wave components, particularly wave I. The relatively shortening in wave I and III latencies in the CLD infants is mainly related to the following two facts at the time of BAER testing: a slightly lower BAER threshold and a slightly higher hearing level at testing in the infants with CLD, compared with the infants without CLD.

In a recent analysis of neurodevelopmental risk factors in premature infants, Borenstein-Levin and colleagues noticed that CLD could be associated with accelerated brainstem auditory maturation.^30^ In the CLD infants, the I‒III interval tended to be shorter than in the non-CLD infants and was significantly shorter at the highest click rate of 91/s. This relatively shortening in the I‒III interval reflects relatively faster auditory conduction and moderately accelerated or advanced maturation in the peripheral or caudal brainstem regions. Previous studies showed that intrauterine stress, such as maternal hypertension and preeclampsia could accelerate neuromotor maturation, and shorten BAER wave V latency and I‒V interval.^31^^,^^32^ The moderately accelerated maturation in the peripheral or caudal brainstem regions in the CLD infants might be mainly related to the chronic sublethal hypoxia during the course of CLD, which acts as a ‘stress’ to stimulate and accelerate the maturation of caudal brainstem regions. The moderate acceleration might be an adaptive change of the caudal brainstem regions to the stress produced mainly by chronic hypoxia and some other associated perinatal conditions. This adaptive change is not clearly shown at term age, but, with the increase in age, is getting clearer.

In the last decades, the BAER has been widely used in pediatric, particularly neonatal, neurology to assess auditory function and detect neuropathology that may involve the brainstem auditory pathway.^11^^,^^12^^,^^33^, ^34^, ^35^ As the most commonly and widely used BAER variable to reflect general brainstem auditory function, the I‒V interval is the sum of the I‒III and III‒V intervals. In the previous study of CLD infants at PCA 40 weeks, i.e., at term, the I–V was significantly increased.^10^ This increase resulted from the combination of a relatively normal I‒III interval and a significant increase in the III‒V interval. In the present study at PCA 50 weeks, the CLD infants showed a slightly shorter I‒III interval but a longer III‒V interval. As a result, the I‒V interval did not show any obvious changes.

There were small differences between the CLD and non-CLD groups in the amplitudes of BAER waves I, III, and V. But none of the amplitudes differed significantly between the two groups of infants at any click rate. The same was true of the V/I and V/III amplitude ratios. Therefore, there is no significant difference between VLBW infants with CLD and those without CLD in the amplitudes of BAER wave components. Neonatal CLD does not significantly affect the amplitudes of BAER wave components, and the neural origins of BAER wave amplitudes are not significantly affected by neonatal CLD.

Taken together, the main BAER difference at PCA 50 weeks between VLBW infants with neonatal CLD and those without CLD infants was a significant increase in the III-V interval in the CLD infants. This abnormality is indicative of delayed auditory conduction at more central brainstem regions in CLD infants. After term, brainstem auditory function is still adversely affected by CLD. Monitoring post-term changes in brainstem auditory function is required to provide valuable information for post-term care and management of CLD infants to help improve neurodevelopmental outcomes.

## Funding

No funding was involved in this study.

## CRediT authorship contribution statement

**Ze Dong Jiang:** Supervision, Investigation, Formal analysis, Writing – original draft. **Cui Wang:** Validation, Data curation, Formal analysis. **James K. Jiang:** . **Jin Wang:** Visualization.

## Conflicts of interest

The authors declare no conflicts of interest.
